# Supplementary material for: The Consumption of Alcoholic Beverages and the Prevalence of Cardiovascular Diseases in Men and Women: A Cross-Sectional Study
Source: Nutrients. 2019 Jun 12;11(6):1318. doi: 10.3390/nu11061318 (PMC6628509; doi:10.3390/nu11061318)
Supplement: Supplementary file 1 [file nutrients-11-01318-s001.pdf]

Table S1. Unadjusted odds ratios and 95% confidence intervals for cardiovascular diseases in men and women.

CVD	Hypertension		Stroke		Coronary disease		Circulatory failure	
Men								
alcohol consumption	OR (95% CI)	p	OR (95% CI)	p	OR (95% CI)	p	OR (95% CI)	p
abstainers (ref.)	1.00		1.00		1.00		1.00	
0.1-10.0 g	1.02 (0.84-1.23)	0.810	0.69 (0.39-1.21)	0.201	0.59 (0.45-0.77)	<0.001	0.73 (0.51-1.06)	0.099
10.1–20.0 g	0.88 (0.70-1.10)	0.257	0.51 (0.24-1.07)	0.076	0.48 (0.34-0.68)	<0.001	0.62 (0.39-0.99)	0.046
20.1-30.0 g	0.63 (0.46-0.85)	0.003	0.31 (0.09-1.08)	0.067	0.25 (0.14-0.46)	<0.001	0.25 (0.10-0.60)	0.002
>30.0 g	0.74 (0.48-1.14)	0.177	0.14 (0.02-1.02)	0.052	0.05 (0.01-0.34)	0.002	0.70 (0.29-1.69)	0.431
Women								
abstainers (ref.)	1.00	p	1.00	p	1.00	p	1.00	p
0.1-5.0 g	0.67 (0.59-0.75)	<0.001	0.42 (0.29-0.62)	<0.001	0.51 (0.43-0.61)	<0.001	0.78 (0.63-0.97)	0.024
5.1–10.0 g	0.51 (0.42-0.62)	<0.001	0.29 (0.12-0.70)	0.005	0.38 (0.27-0.54)	<0.001	0.54 (0.37-0.81)	0.028
10.1-15.0 g	0.37 (0.25-0.54)	<0.001	0.44 (0.11-1.84)	0.261	0.24 (0.11-0.56)	0.001	0.36 (0.14-0.90)	0.028
>15.0 g	0.49 (0.35-0.70)	<0.001	0.20 (0.03-1.49)	0.117	0.31 (0.15-0.63)	0.001	0.62 (0.31-1.25)	0.181

OR – odds ratio; CI - confidence intervals; ref. – reference level
